# The rs1634330 Polymorphisms in the *SOST* Gene Are Associated with Body Composition in Chinese Nuclear Families with Male Offspring

**DOI:** 10.1155/2021/6698822

**Published:** 2021-05-08

**Authors:** Luyue Qi, Lianyong Liu, Li Li, Weiwei Hu, Wenzhen Fu, Ji Hu, Youjia Xu, Zhenlin Zhang

**Affiliations:** ^1^Shanghai Clinical Research Center of Bone Disease, Department of Osteoporosis and Bone Diseases, Shanghai Jiaotong University Affiliated Sixth People's Hospital, Yishan Road 600, Shanghai 200233, China; ^2^Department of Endocrinology and Metabolism, The Second Affiliated Hospital of Soochow University, Suzhou 215004, China; ^3^Department of Endocrinology, Punan Hospital of Pudong New District, No.279 Linyi Road, Pudong, Shanghai 200125, China; ^4^Department of Orthopaedics Surgery, The Second Affiliated Hospital of Soochow University, Suzhou, 215004, China

## Abstract

**Objective:**

The purpose of this study was to explore the effect of the *SOST* gene polymorphisms on body composition in Chinese nuclear families with male offspring.

**Methods:**

1,016 individuals were recruited from 335 Chinese nuclear families with male offspring. The nuclear families consist of at least one male offspring aged 18 to 44. We genotyped the 10 tagged single-nucleotide polymorphisms (SNPs) in *SOST* gene (rs7220711, rs865429, rs851057, rs1708635, rs2023794, rs1234612, rs74252774, rs1634330, rs851058, and rs1513670) in all the above people. We used dual-energy X-ray absorptiometry to measure the composition of the human body. The quantitative transmission disequilibrium test (QTDT) was used to analyze the associations of the SNPs with the body composition.

**Results:**

QTDT analysis showed that rs1634330 was significantly associated with trunk LM (*P* < 0.05). However, haplotypes were not found to be significantly associated with the body composition in the within-family association. The 1000 permutations were consistent with these within-family association results.

**Conclusions:**

Our results showed that the genetic variation in the *SOST* gene may contribute to variations in the body composition of Chinese male offspring.

## 1. Introduction

The *SOST* gene is located on the long arm of human chromosome 17 (17q12–q21), encoding the sclerostin protein of 213 amino acids which negatively regulates bone formation through the Wnt pathway [1, 2]. Loss of function mutations of the *SOST* gene in human can lead to sclerosteosis or Van Buchem disease, an autosomal recessive disease with abnormal high bone mass [3, 4]. In contrast, *SOST* overexpressing mice had a low bone mass phenotype [5]. And pharmacological inhibitors (Romosozumab, etc.) of sclerostin can promote bone formation and inhibit bone resorption in postmenopausal women and men with osteoporosis [6, 7].

The body composition is composited of fat mass (FM) and lean mass (LM), which can be measured by dual-energy X-ray absorptiometry (DXA). In recent years, many studies focused on the association of body composition and Wnt pathway genes. The single-nucleotide polymorphisms (SNPs) of the *LRP5* gene are not an important genetic marker contributing to body composition in Chinese and Caucasian young adults [8, 9]. Our previous study found evidence of an association between body composition and *CTNNB1*and *WNT5B* gene [10]. In the *SOST* gene, the rs10534024 is associated with body composition in Danish young men [11], but rs4792909, rs851054, and rs2023794 are not in Caucasian young men [8]. So, we conducted family-based [quantitative transmission disequilibrium test (QTDT)] studies of 10 tag single-nucleotide polymorphisms (SNPs) in the *SOST* gene to ascertain the effect of *SOST* genetic variations on FM and LM in Chinese nuclear families with male offspring.

## 2. Materials and Methods

### 2.1. Subjects

The study was approved by the Independent Ethics Committee of the Shanghai Jiao Tong University Affiliated Sixth People's Hospital. All the subjects involved in this study signed informed consent documents before entering the project. The recruited subjects were the local population of Shanghai City on the Middle East Coast of China by the Department of Osteoporosis and Bone Diseases. The inclusion and exclusion criteria in this study were detailed as before [12]. From 2004 to 2007, 349 nuclear families from Chinese Han nationality were recruited, including both parents, with a total of 1058 people. The nuclear family has at least one male offspring with an average age of 18–44 years. The age, gender, medical history, and family history of each subject were recorded.

### 2.2. Body Composition Measurements

All subjects underwent body composition measurements with a lunar prodigy DXA densitometer (GE Healthcare, Madison, WI, USA). Such a tool allows for the evaluation of fat mass (FM) (kg) and lean mass (LM) (kg) (including arms, legs, trunk, and total body) by conducting by the same well-trained technologist throughout the study. The method of machine calibration and the coefficient of variation (CV) of DXA in measuring lean meat and fat were the same as before [12, 13]. Height and body weight were estimated using standardized equipment. BMI was estimated by dividing the weight in kilograms by the square of the height in meters. The percentage of fat mass (PFM) and the percentage of lean mass (PLM) were estimated as the ratio of fat mass and lean mass to body weight, respectively.

### 2.3. SNP Selection

The candidate SNPs in the *SOST* gene were evaluated as before [14]. The recruitment criteria were as follows: (1) validation status, especially in Chinese; (2) minor allele frequencies >0.05; (3) pairwise linkage disequilibrium (LD) exceeds a threshold *r*^2^ of 0.8; and (4) classification as tag SNPs. Hence, 10 tag SNPs were selected which are located in 3' flanking (rs7220711 and rs1513670), intron 2 (rs865429), and 5' flanking (rs1234612, rs851058, rs1634330, rs1708635, rs74252774, rs2023794, and rs851057), respectively.

### 2.4. Genotyping

Genomic DNA was obtained from peripheral venous blood samples of every participant and genotyped the 10 tag SNPs. Using a SNaPshot SNP genotyping technique to genotype the SNPs. Primers were designed by online Primer3 software (http://bioinfo.ut.ee/primer3-0.4.0/). 10 pairs of PCR primers were designed to amplify 10 fragments of 136–241 bp in 10 loci, and 10 extended primers adjacent to the SNP loci were designed for single-base extension (the primer sequence is shown in Table 1). The PCR products were obtained by multiplex PCR using the HotStarTaq (Qiagen). After being purified by the shrimp alkaline enzyme (SAP, Promega) and the exosome I (Epicentre), the PCR products were extended by SNaPshot Multiplex kit (ABI). The extended products were purified by SAP and then sampled on ABI 3730 XL. SNP genotyping was analyzed with GeneMapper 4.1 (Applied Biosystems).

### 2.5. LD and Haplotype Analyses

In this study, the Haploview program (version 4.2) was used to generate Linkage disequilibrium (LD) plots of SNPs from SNP genotyping data [15]; D' represented the degree of LD of the SNPs in the *SOST* gene. Haplotypes were constructed as mentioned above [14]. The frequencies of the genotypes and haplotypes were calculated using a group of unrelated parents of nuclear families.

### 2.6. Statistical Analyses

Allele frequencies were evaluated by simple counting. Hardy–Weinberg equilibrium (HWE) was calculated by a *χ*^2^ goodness-of-fit statistical test. The orthogonal model in the QTDT program was used to test for population stratification, linkage, and the within-family association between SNPs and haplotypes and body composition phenotypes. The QTDT software package is available online on the Internet (http://csg.sph.umich.edu/abecasis/QTDT/). In this model, founder and sibling genotypes are included in the within-family and the trio-based transmission disequilibrium test (TDT) is extended to quantitative trait data. The BMI was adjusted by age as a covariate. Gender was not used as a covariate because all the offspring were males. We only calculated the male offspring of the nuclear families and parents' phenotypes were excluded in the QTDT. To avoid false-positive results generated in multiple tests in our study, permutations (1000 simulations) were performed to produce empirical *P* values [16–18]. All analyses considered that *P* < 0.05 was statistically significant.

After adjusting for age, the proportions of the variation in body composition of unrelated sons were obtained from the general linear model-ANOVA (GLM-ANOVA) explained by the SNPs. Statistical analysis was performed using the SPSS version 25.0 (SPSS Inc. of IBM, USA).

## 3. Results

### 3.1. Basic Characteristics of Study Subjects

Because of the poor quality of DNA, 7 subjects from 7 nuclear families were excluded, and 7 sons from 7 nuclear families deviated from Mendelian inheritance when initial QTDT analysis showed. So, the 14 families were removed from the study. Finally, we recruited 1,016 subjects from 335 male offspring nuclear families, including 670 parents and 346 sons. The basic characteristics of the subjects are reported in Table 2.

### 3.2. Tag SNPs Genotyping and Haplotypes Frequency

10 tag SNPs in the *SOST* gene were genotyped, and none were excluded from the further analysis. Each of the tag SNPs had a minor allele frequency (MAF) > 0.05 and was in Hardy–Weinberg equilibrium (HWE) (Table 3).

**Figure 1:**
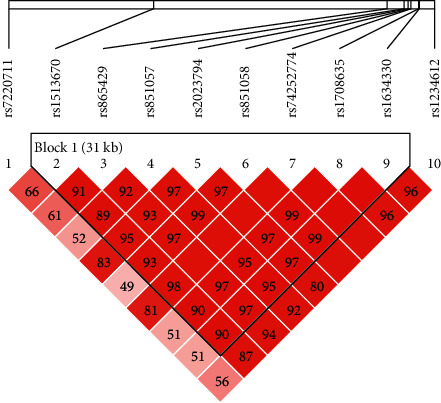
Linkage disequilibrium (LD) patterns for the *SOST* gene. The pairwise LD values (D' × 100) of all SNPs are given in each diamond. D' is calculated as D divided by the theoretical maximum for the observed allele frequencies. Bright red filled diamonds without values indicate a D' = 1. One block with high LD was identified. Numbers in bracket indicate the length of the block.

We calculated one block with the size of 31 kb of substantial LD in the *SOST* gene using Haploview (Figure 1). The common haplotypes for the block were represented by rs1634330, rs74252774, rs851057, rs2023794, rs851058, rs865429, rs1708635, and rs1513670. The 8 haplotypes accounted for 94.9% of the 670 unrelated parents (Table 4).

### 3.3. Association between Tag SNPs and Haplotypes with Body Composition Variations

The association between obesity-related phenotypes and single tag SNP in *SOST* was performed using QTDT for 335 male offspring nuclear families. There were 237, 243, 204, 238, 91, 228, 82, 230, 230, and 160 informative nuclear families for the TDT analysis at rs7220711, rs865429, rs851057, rs1708635, rs2023794, rs1234612, rs74252774, rs1634330, rs851058, and rs1513670, respectively. TDT results showed that population stratification were found for rs7220711 and arms FM (*p*=0.0048), trunk FM (*p*=0.0254), legs FM (*p*=0.0092), and total LM (*p*=0.0172); rs1513670 and arms and trunk FM (*p*=0.0165 and 0.0070, respectively); and rs74252774 and total FM (*p*=0.0303). The within-family associations were found for rs7220711 and total FM (*p*=0.0080); rs1513670 and legs and total FM (*p*=0.229 and 0.0070, respectively); rs851057 and arms, trunk and total FM (*p*=0.0205, 0.0054 and 0.0141, respectively), and rs1634330 and trunk LM (*p*=0.0049). In order to avoid the error caused by multiple parameters tests, we performed the permutation 1,000 tests. The association of rs1634330 and trunk LM (*p*=0.0410) was found after the permutations (Table 5).

For haplotype-based association analyses, 231, 188, 186, 74, 43, and 44 informative nuclear families at TAGTGGAT, TGGTGGAT, CACTAGTC, CACCATTC, CACTGGAT, and CAGTGGAT in the block were evaluated for the TDT analysis. However, we failed to found significant associations between any haplotypes and body composition in the permutation 1,000 tests (Table 6).

Besides, after adjusting for age as a covariate, we used ANOVA to obtain the proportion of changes in body composition explained by rs1634330. The rs1634330 explained 0.26% of the total variation in trunk LM.

## 4. Discussion

In our study, we measured the FM and LM of arms, trunk, legs, and total body and genotyped the 10 tag SNPs in the *SOST* gene of 335 male offspring nuclear families with 1,016 subjects from the Han ethnic group of China. We used QTDT to observe the association between polymorphisms in the *SOST* gene and body composition. We observed a significant within-family association between rs1634330 and the trunk FM. The 1000 permutations that were subsequently simulated were consistent with these within-family association results. We deem that the research of nuclear families is a better interpretation of the association between the polymorphisms in the *SOST* gene and body composition.

Given the above results of within-family association, the statistical power of our study sample to calculate a quantitative trait locus (QTL) in the TDT should be considered. First of all, the sample was composed of 335 nuclear families in China, including 670 parents and 346 sons. Family-based association analysis can avoid the impact of population stratification [19]. In addition, the heterozygosis of participants in our sample was high (MAF of 10 SNPs was > 0.06). On the whole, QTDT can use all the information in a pedigree to conduct powerful tests of association that are robust in the presence of stratification (http://csg.sph.umich.edu/abecasis/QTDT/). Finally, in order to evaluate the false positive and negative results caused by the multiple tests, 1,000 permutations were simulated. Although there was no statistical significance for the within-family association between single haplotype in *SOST* and body composition in this study, on account of our previous findings [10, 20], it can be said with certainty that our study sample is adequate to evaluate the potential QTL influencing on body composition variation; the results of this study should be more reliable because of the robustness of the TDT approach.

So far, two studies investigated the association between the polymorphism in the *SOST* gene and body composition [8, 11]. We observed the association between rs1634330 and the trunk FM, in line with the findings of Piters et al. in Danish men from the Odense Androgen Study cohort. On the contrary, the study reported a lack of association between *SOST* polymorphisms and body composition in Caucasian adults from five different academic centers of Granada (Spain). The same to us is that they all choose young men as research objects and select *SOST* gene in body composition variations as study aim. But it is necessary to note that these studies are rather heterogeneous concerning the object ancestry (Chinese, Caucasian), gender (males and females), age (young and old), measurement methods of body composition (DXA, body composition analyzer, and light clothing), SNPs selection (tagged SNPs, reported previous association of body composition), and statistical method (QTDT, linear regression analysis, and multiple linear regression analysis).

Recent data [21] showed that *SOST*^*-/-*^ mice have less body fat and smaller adipocytes, accompanied by improved glucose tolerance and enhanced insulin sensitivity. Contrarily, *SOST* overexpression mice demonstrated excess adipose tissue and impaired glucose handling. In *SOST*^*-/-*^ mice white adipose tissue, Wnt signaling markers were increased and Bmp signaling indicators were reduced. In vitro and in vivo, Bmp4 and its receptor Bmpr1a levels were negatively correlated with the Wnt signaling levels in adipocytes. In vitro, Bmp antagonist noggin could abolish the effects of recombinant sclerostin on adipocyte metabolism. The above data showed that sclerostin indirectly regulated Bmp signaling to exhibit its effects on adipocyte metabolism. Osteocytes can stimulate myogenesis and strengthen muscle contractile function likely via the Wnt/*β-*catenin signaling. In the above progress, sclerostin inhibited muscle function by depressing both the effects of osteocyte-like cells and WNT3a on myoblasts differentiation [22]. The rs1634330 is located in the 5' flanking which is next to the 5' end of *SOST*. This region includes the promoter and may include enhancers or other protein binding sites. The primary function of this region is the regulation of gene transcription [23]. Further study is required to confirm whether rs1634330 might affect the binding of other proteins or regulation of gene transcription of *SOST*.

Of course, in our study, we also have several limitations. First, our nuclear families are small sample size that contained few sibling pairs and have only two generations, which lowered the power of the linkage analysis; second, the selection of SNPs was not based on functionality. We select a group of 10 tagged SNPs of the *SOST* gene that is mostly within a single large LD block, which results in less association between the *SOST* gene and body composition.

To our knowledge, this is the first time to study the relationship between the *SOST* gene polymorphism and the variation of body composition in the core family of male offspring. We found the genetic associations between the *SOST* gene and the body composition of Chinese male offspring. The polymorphisms of rs1634330 in the *SOST* gene may contribute to variations in trunk LM of Chinese young men, but only 0.26% of the total variation in trunk LM (the coefficient of variation of the DXA measurements for LM is 1.12%) was explained by rs1634330. Additional research in larger sample populations and SNPs of the *SOST* gene with high putative functionality is warranted to ascertain the role of the *SOST* gene in body composition variations.

## Figures and Tables

**Table 1:** Primers used for PCR.

Tag SNPs	Primers (forward/reverse)	Extended primers
rs7220711	(F) 5'-AGGAGCAGCTGCAAGGAAGACA-3'	(F) 5'-TTTTTTTTTTTTAGTTCCCATTTAGTATAAAAGCTGGCTC-3'
	(R) 5'-CCACCCTAGGCCCTGAAATAGC-3'	
rs1513670	(F) 5'-TGGCAACAGTGGCAGCTACAA-3'	(F) 5'-GCCCCACATGCCAGGACAC-3'
	(R) 5'-TCTAATGCTCCCCATTCCTCTCC-3'	
rs865429	(F) 5'-GGAATGAGGCAAGGTTGGGACT-3'	(R) 5'-TTTTTTTTTCCTGCAGTGTGCATTGCCCA-3'
	(R) 5'-TGGGGGACAGATCTCCACAAAG-3'	
rs851057	(F) 5'-GCCTTGGCCCTGCATATAATGA-3'	(R) 5'-TTTTTTTTTTTTTTTTTTTTTTTTTTTCCCCCACGCCTCTACCTGC-3'
	(R) 5'-AGGATCTGGGCAGCCTCTTCAC-3'	
rs2023794	(F) 5'-CCTTCAGGGCAGATGAAAACAGC-3'	(F) 5'-TTTTTTTTTTTTGATGAAAACAGCTGTGGCCATTGT-3'
	(R) 5'-GCAAGAAGGCAGTCGTCTGGTC-3'	
rs851058	(F) 5'-CCAGCAGAGCCGGTAGTGTTGT-3'	(R) 5'-TTTTTTTTTTTTTTTTTTTAGGGTCATAGACAAGGGGAGGTGG-3'
	(R) 5'-GCAAGAAGGCAGTCGTCTGGTC-3'	
rs74252774	(F) 5'-AAAGGAGGGGTGACTGCAGGAT-3'	(R) 5'-TTTTTTTTTTTTTTTTTTTTTTTTTTTTTGGAAAGGAAAGGAAAGGAAATCACGT-3'
	(R) 5'-CTGAGCCATTCAGAGGGGTGTG-3'	
rs1708635	(F) 5'-CGAGGCTGCAGTGAGCCATAC-3'	(R) 5'-TTTTTTTTTTTTTTTTTTTTTTTTTTTGAGAYGGGGTCTCACTCTGTCA-3'
	(R) 5'-GTTTCGCAAGGTGTTATTGTTGTGG-3'	
rs1634330	(F) 5'-GGAAGGAGGTGGGCAACAGG-3'	(R) 5'-TGTGCACGCACACAGTAGAGGTTAA-3'
	(R) 5'-TGACACAATTTACCAATCTTACCCACA-3'	
rs1234612	(F) 5'-CCCAGCCGATTTTTTTAAACATTGA-3'	(R) 5'-TTTTTTTTATTAAACGTTTGGCGAGTGAACATC-3'
	(R) 5'-TGGTATGCGAGCTCCTGGAGAG-3'	

**Table 2:** Basic characteristics of the subjects (mean ± SD).

Characteristics	Father (*n* = 335)	Mother (*n* = 335)	Son (*n* = 346)
Age (years)	60.8 ± 7.1	58.2 ± 6.4	29.5 ± 6.1
Height (cm)	167.8 ± 6.0	155.8 ± 5.6	172.9 ± 6.1
Weight (kg)	69.5 ± 9.6	58.0 ± 8.1	70.5 ± 10.3
BMI (kg/m^2^)	24.7 ± 3.1	23.9 ± 3.0	23.6 ± 3.2
Arms FM (kg)	—	—	1.254 ± 0.718
Trunk FM (kg)	—	—	9.250 ± 4.275
Legs FM (kg)	—	—	4.661 ± 1.855
Total FM (kg)	—	—	15.750 ± 6.734
PFM (%)	—	—	21.66 ± 7.02
Arms LM (kg)	—	—	5.778 ± 0.783
Trunk LM (kg)	—	—	24.264 ± 2.736
Legs LM (kg)	—	—	17.372 ± 2.206
Total LM (kg)	—	—	51.364 ± 5.543
PLM (%)	—	—	73.51 ± 6.74

**Table 3:** Information of the analyzed *SOST* tag SNPs.

Tag SNP	Domain	Polymorphism	Physical position	HWE	MAF in dbSNP (CEU)	MAF in dbSNP (CHB)	MAF in this study
rs7220711	3' flanking	G/A	41789965	0.827	G:0.384	G:0.272	G:0.297
rs1513670	3' flanking	C/T	41807331	0.278	C:0.616	C:0.388	C:0.374
rs865429	Intron	G/A	41835215	0.953	G:0.121	G:0.248	G:0.278
rs851057	5' flanking	C/G	41837264	0.935	C:0.884	C:0.364	C:0.357
rs2023794	5' flanking	C/T	41837660	0.242	C:0.040	C:0.087	C:0.074
rs851058	5' flanking	A/G	41837719	0.333	A:0.364	A:0.364	A:0.306
rs74252774	5' flanking	G/T	41838012	0.415	G:1.000	G:0.918	G:0.934
rs1708635	5' flanking	T/A	41838894	0.276	T:0.606	T:0.296	T:0.317
rs1634330	5' flanking	C/T	41839069	0.249	C:0.606	C:0.296	C:0.317
rs1234612	5' flanking	C/T	41840802	0.406	C:0.303	C:0.141	C:0.169

SNP: single-nucleotide polymorphism; HWE: Hardy–Weinberg equilibrium; MAF: minor allele frequency; and dbSNP: SNP database.

**Table 4:** The structure and frequency of *SOST* haplotypes for all available SNPs.

Index	rs1513670	rs1634330	rs1708635	rs2023794	rs7220711	rs74252774	rs851057	rs851058	Frequency
1	T	A	G	T	G	G	A	T	0.336
2	T	G	G	T	G	G	A	T	0.247
3	C	A	C	T	A	G	T	C	0.224
4	C	A	C	C	A	T	T	C	0.066
5	C	A	C	T	G	G	A	T	0.039
6	C	A	G	T	G	G	A	T	0.037

**Table 5:** *P* values of tests for population stratification, total association, and within-family association in *SOST* tag SNPs using QTDT.

	rs7220711	rs1513670	rs865429	rs851057	rs2023794	rs851058	rs74252774	rs1708635	rs1634330	rs1234612
*Tests of population stratification*										
BMI	0.7074	0.6894	0.7586	0.7587	0.8942	0.4900	0.7200	0.4716	0.4893	0.8076
Arms FM	**0.0048**	**0.0165**	0.2552	0.0732	0.6766	0.6049	0.9380	0.7242	0.7303	0.6490
Trunk FM	**0.0254**	**0.0070**	0.1334	0.0341	0.6383	0.4957	1.000	0.6549	0.6644	0.4218
Legs FM	**0.0092**	0.0580	0.0507	0.1997	0.4166	0.7020	0.6692	0.7908	0.8235	0.1418
Total FM	0.1185	0.1368	1.0000	0.0554	0.2569	1.0000	**0.0303**	1.0000	0.8551	0.6295
PFM	0.1153	0.3676	0.9686	0.7228	0.6640	0.8898	0.7927	0.9960	0.9757	0.4735
Arms LM	0.1239	0.4489	0.6243	0.7506	0.9115	0.3548	0.9985	0.4969	0.5412	0.9973
Trunk LM	0.5131	1.0000	0.2753	1.0000	0.4952	1.0000	1.0000	1.0000	0.7838	1.0000
Legs LM	0.1240	0.4516	0.8182	0.9848	0.7818	0.8764	1.0000	0.7451	0.7795	0.8853
Total LM	**0.0172**	0.1939	0.5489	0.7441	0.3545	0.2245	1.0000	0.2848	0.4609	1.0000
PLM	0.3937	0.9953	0.7482	0.6091	0.6667	0.8943	0.8248	0.9387	0.9776	0.4766

*Tests of total association*										
BMI	0.1142	0.2459	0.3638	0.2068	0.4186	0.4013	0.4467	0.4696	0.4545	0.5933
Arms FM	0.4535	0.0842	0.8849	0.0968	0.7375	0.6908	0.9506	0.5982	0.5941	0.6581
Trunk FM	0.4794	**0.0305**	0.5984	**0.0399**	0.6767	0.4312	0.8598	0.3854	0.3807	0.9208
Legs FM	0.8614	0.1845	0.7668	0.2279	0.8633	0.9596	0.9311	0.8576	0.8532	0.7517
Total FM	0.3567	0.0697	0.6320	0.1024	1.0000	0.7282	1.0000	0.6995	0.6738	0.8949
PFM	0.9585	0.2622	0.4958	0.6412	0.9750	0.7642	0.7684	0.9840	0.9939	0.1933
Arms LM	0.9474	0.3626	0.7602	0.6595	0.7027	0.8896	0.8348	0.7267	0.7276	0.6528
Trunk LM	0.5472	0.5204	1.0000	1.0000	1.0000	0.9470	0.7455	0.6553	0.6528	0.2226
Legs LM	0.9256	0.3803	0.6209	0.7734	0.8946	1.0000	0.9760	0.7943	0.7841	0.2845
Total LM	0.5000	0.1247	0.5408	0.5108	0.9062	0.9663	0.9218	0.5606	0.5544	**0.0216**
PLM	0.5649	0.6311	0.5855	0.8600	1.0000	0.7001	0.7971	0.9627	0.9678	0.1491

*Tests of within-family association*										
BMI	0.2775	0.3756	0.9067	0.3953	0.7896	0.8153	0.5033	0.7473	0.7760	0.6544
Arms FM	**0.0042**	**0.0038**	0.3213	**0.0205**	0.8167	0.5253	0.9231	0.5924	0.5958	0.8017
Trunk FM	**0.0207**	**0.0008**	0.1091	**0.0054**	0.7916	0.3342	1.0000	0.4481	0.4470	0.4615
Legs FM	**0.0157**	**0.0229**	0.0556	0.0954	0.5109	0.7119	0.6709	0.7524	0.7790	0.2183
Total FM	**0.0080**	**0.0070**	0.2781	**0.0141**	0.6715	0.6309	1.0000	0.6646	1.0000	1.0000
PFM	0.2011	0.1732	0.7028	0.5826	0.7312	0.9657	0.7029	1.0000	0.9755	0.2026
Arms LM	0.2015	0.2661	0.7754	0.6197	0.9173	0.3837	0.9144	0.4451	0.4820	0.6600
Trunk LM	0.7724	0.4325	0.6181	0.6255	0.4947	0.6238	0.7352	0.4396	**0.0049**	0.3835
Legs LM	0.2007	1.0000	0.9831	0.8793	0.8470	0.6209	0.9872	0.6821	0.6998	0.5455
Total LM	0.0860	0.0588	0.6200	0.5459	0.4581	0.2041	0.9568	0.2234	0.2579	0.1249
PLM	0.6961	0.8008	1.0000	0.5987	0.7195	0.9266	0.7445	0.9672	1.0000	0.1814

*P 1000 permutation of within-family association*										
BMI	0.2930	0.3870	0.9170	0.4040	0.7880	0.8320	0.5220	0.7390	0.7690	0.6740
Arms FM	0.0740	**0.0410**	0.5470	0.1040	0.8630	0.6970	0.9530	0.7170	0.7170	0.8840
Trunk FM	0.1560	**0.0230**	0.2910	0.0680	0.8010	0.5500	0.9330	0.6450	0.6430	0.6430
Legs FM	0.0930	0.0890	0.1750	0.2150	0.6300	0.8000	0.7640	0.8260	0.8430	0.3940
Total FM	0.1270	0.0860	0.4170	0.1090	0.6320	0.6410	0.7510	0.6360	0.8170	0.8820
PFM	0.1310	0.930	0.6530	0.5470	0.7100	0.9670	0.6830	1.0000	0.9730	0.1780
Arms LM	0.2080	0.2420	0.7770	0.630	0.9280	0.3950	0.9230	0.4550	0.4980	0.6880
Trunk LM	0.7130	0.3860	0.5700	0.5840	0.4640	0.5890	0.7010	0.4390	**0.0410**	0.3990
Legs LM	0.1650	0.9920	0.9340	0.8440	0.7990	0.6050	0.9050	0.6580	0.6730	0.5350
Total LM	0.3040	0.2440	0.7130	0.6730	0.6310	0.4560	0.8770	0.4700	0.5040	0.4050
PLM	0.6130	0.7240	0.9950	0.4860	0.6710	0.8940	0.7070	0.9540	0.9990	0.1000

BMI: body mass index; QTDT: quantitative transmission disequilibrium test; FM: fat mass; LM: lean mass; PFM: percentage of FM; and PLM: percentage of LM. BMI and body composition phenotype values are adjusted for age. Bold numbers indicate significant *p* values (*p* < 0.05).

**Table 6:** *P* values of tests for population stratification, total association, and within-family association in *SOST* haplotypes using QTDT.

	CACTGGAT	CACTAGTC	CACCATTC	CAGTGGAT	TGGTGGAT	TAGTGGAT
*Tests of population stratification*						
BMI	0.1345	0.7036	0.6155	0.5591	0.8234	0.9163
Arms FM	0.0785	0.3957	0.2127	0.0892	0.5264	0.0933
Trunk FM	0.0568	0.3232	0.2205	0.0973	0.3628	0.0644
Legs FM	0.2020	0.8096	0.0718	0.0677	0.0967	0.4443
Total FM	**0.0434**	0.5896	0.1747	0.5626	0.0895	1.0000
PFM	0.4952	0.7563	0.3280	0.4137	0.9272	0.2319
Arms LM	0.0987	0.1793	0.4772	0.3501	0.3760	0.1518
Trunk LM	**0.0303**	0.0728	0.4102	0.6748	1.0000	0.1411
Legs LM	0.1733	0.4275	0.4275	0.2240	0.5944	0.2561
Total LM	0.0730	0.0845	0.1240	**4e-005**	1.0000	0.0531
PLM	**0.0365**	0.8282	0.4909	0.5839	0.7417	0.6347

*Tests of total association*						
BMI	0.3953	0.6183	0.3097	0.9612	0.3564	0.0512
Arms FM	**0.0105**	0.3659	0.9312	0.9970	0.7207	0.0906
Trunk FM	**0.0048**	0.1507	0.8193	1.0000	0.8968	0.1262
Legs FM	**0.0072**	0.6696	0.9652	0.7794	0.9623	0.4744
Total FM	**0.0164**	1.0000	0.7718	0.7693	1.0000	0.1930
PFM	0.1041	0.7912	0.6941	0.7276	0.8148	0.9042
Arms LM	0.2736	0.3777	0.5566	0.7820	0.8393	0.7380
Trunk LM	1.0000	1.0000	1.0000	0.9348	1.0000	1.0000
Legs LM	0.2736	0.5674	0.6738	0.7456	1.0000	0.9580
Total LM	**0.0299**	0.2871	0.3573	1.0000	0.8362	0.8887
PLM	0.4696	0.9138	0.5649	0.8140	0.8730	0.6295

*Tests of within-family association*						
BMI	0.0857	0.9137	0.3587	0.6015	0.5560	0.3028
Arms FM	**0.0087**	0.2485	0.2746	0.1440	0.6497	0.0243
Trunk FM	**0.0041**	0.1300	0.3120	0.2244	0.3492	0.0193
Legs FM	**0.0254**	0.6888	0.1074	0.1533	0.1195	0.3160
Total FM	**0.0105**	0.3215	0.2122	0.2295	0.6707	0.0204
PFM	0.7896	0.6866	0.5433	0.3850	0.9724	0.2876
Arms LM	0.3637	0.1087	0.7530	0.3517	0.3786	0.1621
Trunk LM	1.0000	0.9068	1.0000	0.2177	1.0000	0.0386
Legs LM	0.4767	0.3316	0.6029	0.2354	0.6333	1.0000
Total LM	0.1135	**0.0430**	0.3482	0.0748	0.6653	0.1095
PLM	0.1585	0.8092	0.7888	0.5596	0.8334	0.8953

*P 1000 permutation of within-family association*						
BMI	0.1220	0.9080	0.4040	0.6430	0.5910	0.3360
Arms FM	0.1110	0.4720	0.5300	0.3360	0.7690	0.1490
Trunk FM	0.0820	0.3350	0.5010	0.3850	0.5280	0.1380
Legs FM	0.0561	0.7780	0.2460	0.2990	0.2760	0.5090
Total FM	0.1610	0.4870	0.4040	0.3860	0.7330	0.1410
PFM	0.7910	0.6150	0.4260	0.2010	0.9630	0.2220
Arms LM	0.4700	0.1030	0.7530	0.5680	0.4040	0.1880
Trunk LM	0.8480	0.7370	0.7420	0.1440	0.7540	0.0850
Legs LM	0.4440	0.2780	0.5140	0.1030	0.5810	0.9570
Total LM	0.4310	0.2490	0.5320	0.1830	0.7620	0.3440
PLM	0.1130	0.7580	0.7230	0.5070	0.7970	0.8850

BMI: body mass index; QTDT: quantitative transmission disequilibrium test; FM: fat mass; LM: lean mass; PFM: percentage of FM; and PLM: percentage of LM. BMI and body composition phenotype values are adjusted for age. Bold numbers indicate significant *p* values (*p* < 0.05).

## Data Availability

The data used to support the findings of this study have not been made available because of the restrictions by the ethics in the hospital to protect patients' privacy.
